# Dataset construction to detect human behavior with the help of emotions, sentiments and mood for Roman Urdu

**DOI:** 10.1016/j.dib.2023.109906

**Published:** 2023-12-09

**Authors:** Asia Samreen, Syed Asif Ali

**Affiliations:** a Department of Computer Science, Bahria University, Karachi Campus, 13 National Stadium Road, Karachi 75260, Pakistan; b Department of Computer Science, Sindh Madressatul Islam University, Hasrat Mohani Road, Karachi 74000, Pakistan

**Keywords:** Natural language processing, Bilingual text, Text transliteration, Text annotation, Emotion Lexicons

## Abstract

Roman Urdu and English are often used together as a hybrid language for communication on social media. Because writers don't worry about spelling when utilizing the English alphabet to write Urdu during texting, it becomes challenging to interpret mixed codes for emotions. There are over 14,000 emotion lexicons in this dataset, each of which lists nine different emotions and their polarities. The NRC emotion lexicons [8] provided in Urdu have been transliterated into Roman Urdu. To verify that the provided translation is accurate, we used three online dictionaries of Urdu. A Python script that transliterates words from Urdu to Roman Urdu has been used to develop Roman Urdu transliteration. Sentiment and mood, depending on the emotion lexicon, are also provided. The textual data has been annotated using the unigram feature and distance estimation among strings and lexicons. Approximately 10,000 sentences from the baseline sample have been automatically annotated.

Specifications Table

**Table:** 

Subject	Computer Science: Artificial Intelligence
Specific subject area	Prediction of emotions, sentiments and moods for mixed codes
Data format	Analyzed Data Filtered Data
Type of data	Textual data(Roman Urdu Lexicons) Annotated data
Data collection	A new dataset was created using Urdu lexicons from the multilingual sentiment lexicon collection [1]. To obtain the annotated dataset, raw text was collected from Twitter by writing a script in Python, and then all IDs were removed for the purpose of anonymity. We have also applied filters to select specially bilingual data, whose preferred sentence is one that has Roman Urdu, and English wording. We have also collected some publicly available mixed-coded data to test our script for automatically annotation of text.
Data source location	Bahria University, Karachi, Pakistan Sindh Madersa tul Islam University, Karachi, Pakistan
Data accessibility	Repository name: Mendeley Data identification number(DOI): 10.17632/d5j9fgbdcn.1 Direct URL to data: https://www.data.mendeley.com/datasets/d5j9fgbdcn/2 Instructions for accessing these data: Emotions Lexicons in Roman Urdu, along with Emotion Polarity and Mood categorization”, Mendeley Data, V1

## 1 Value of the Data

• To the best of our knowledge, this is the first dataset for human behavior analysis that represents bilingual text, i.e., Roman Urdu and English for emotion lexicons.
• This dataset consists of two files: one contains emotion lexicons in both languages and serves as a dictionary, while the other file contains annotated data.
• This dataset includes 10,000 tweets that have been annotated with regard to sentiment, mood, and emotion, as well as roughly 14,000 bilingual emotion lexicons.
• The dataset can be used to analyze the text, which may be written entirely in English, entirely in Roman Urdu, or a combination of both languages.
• This dataset can be used to explore sentiments, emotions, and moods to identify bias in behavior by training the machine learning models.

## 2 Data Description

Social networks such as WhatsApp and Twitter provide a friendly environment where users mix native language and English terms during communication. However, due to the use of mixed languages, software systems may generate inaccurate or partial results [1]. States that it takes a lot of resources to create sentiment lexicons for multiple languages or mixed codes. Emotion detection [[2], [3]] plays a vital role in accomplishing several tasks [4], such as behavior recognition, etc. The raw text written in any language must be annotated before using natural language processing algorithms to extract linguistic aspects [5]. Roman Urdu and English texts or textual data have revealed that people often express their anger or happiness in words of their native language, such as “*I have won the match, maza aagaya”,* or “*I never feel like this; don't take it to heart; come on! Chalta hai*”. Here, “*maza aagaya*” and “*chalta hai*” are from the native language, which means “*it was fun*" and “*that's what everyone does*,” respectively. We have prepared the dataset using the analysis of Roman Urdu's linguistic features since the construction of an emotion-detecting lexicon entails a deep analysis of linguistic properties [2,6]. This dataset consists of emotion lexicons in both English and Roman Urdu. The dataset contains nine types of emotions to express behavioral features for some individuals. We have also introduced an “interest” emotion in our dataset that can help predict the behavior or action taken by a person more accurately [8].

This dataset consists of two files, one of which is a Microsoft Excel file in which English and Roman Urdu lexicons for emotions, along with polarity, have been provided. In linguistics, lexicons are collections of terms and specialized vocabulary that are specific to a subject matter and are used to refer to words and phrases. The proposed list of lexicons represents the emotions, their polarity, and the subjective mood relevant to the emotion. For instance, in the mixed-coded sentence “Correctness k se pata chaley?” (How do I know the correctness?) “Correctness” is the lexicon that shows “trust” as an emotion having a positive polarity. In the second file, which is a CSV file, annotated data or an application of the emotion lexicons has been provided.

The dataset contains nine emotions, and we have provided the polarity of each emotion lexicon; for instance, “displeasure” or “narazgi” has a negative polarity, and during a conversation, if someone uses this kind of wording, it means he or she will not be in a happy mood. Moods will be categorized into three groups based on the polarity of the emotion: good, bad, and moderate. Table 1 shows all emotions and categorizations of moods based on polarity.

**Table 1:** Emotions and moods.

S.No	Emotion	Polarity	Mood
1	Anger: *antagonism to a person or entity*	Negative	Bad
2	Anticipation: *excitement over something enjoyable or exciting*	Positive	Good
3	Disgust: a strong dislike for anything objectionable	Negative	Bad
4	Fear: *emotional response to a potentially perilous situation*	Negative	Bad
5	Interest: *an eagerness to learn about someone or something*	Positive	Good
6	Joy: *a feeling of extreme delight and pleasure.*	Positive	Good
7	Sadness: *sentiments of sadness and depression*	Negative	Bad
8	Surprise: *an unforeseen or astounding occurrence, fact, etc*	Mixed	Mixed
9	Trust: *strong faith in someone or something's capacity, truth, or dependability*	Positive	Good

## 3 Experimental Design, Materials and Methods

**Fig. 1:**
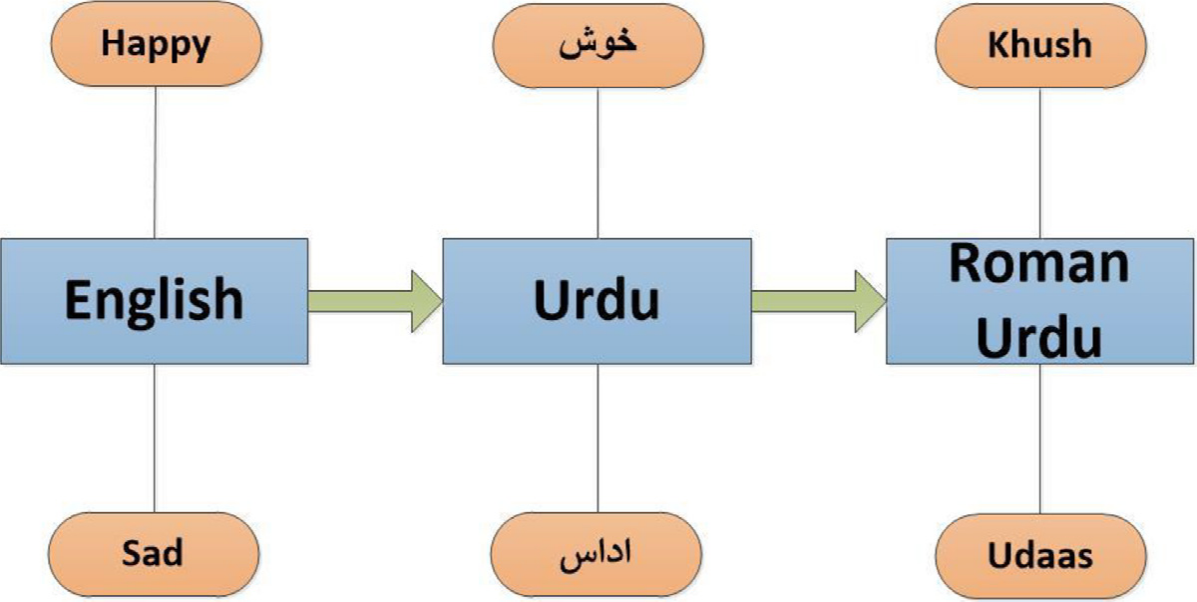
process to get the Roman Urdu lexicons.

To build emotion lexicons, we have used NRC emotions [7] provided for various languages. We have picked all emotion words listed in Urdu and passed the list to function as described in Fig. 1, which returns a list of Roman Urdu for the same Urdu words.

The algorithm which is used to transliterate is given below:

**Table:** 

**Algorithm:**
Dictionary UnitoEngChardic UrdutoRomanurdu (word) For unicode, character in UnitoEngChardic.items() string = string.replace(unicode, character) return string

A dictionary of Unicode for Urdu alphabets has been maintained, along with the corresponding English alphabets. If a word such as “قانون” (law) is input, then it should be translated to “Qanon,” which helps to convert Urdu wording to Roman Urdu easily, as described in Table 2.

**Table 2:** Transliteration process of lexicons.

English	Urdu	Transliteration			
		Urdu character	Corresponding English character/s	UTF-16 (hex)	Python source code
law	قانون	ق	Q	0 × 0642	u"\u0642"
		ا	A	0 × 0627	u"\u0627"
		ن	N	0 × 0646	u"\u0646"
		و	O	0 × 0648	u"\u0648"
		ن	N	0 × 0646	u"\u0646"

The list of emotion lexicons in Roman Urdu has been acquired, but it should also require some corrections to make it standardized. Additional changes are made to the dataset before it is finalized; these modifications are shown below:

1. *Spelling correction:* To correct minor spelling mistakes, the selection and replace method has been employed. For instance  “Kamiabi” (success) will be corrected as “Kamiyabi” by replacing “ia” with “iya”. As mentioned above, some transliterated words contain “o” instead of w," so it has also been corrected manually. For instance, the word “aoam” has been corrected to “awam”. A few words are corrected to entertain “Zair”, Zabar," and “Paish” or circumflex or process the special phonetic sounds. In Table 3, a few examples of such corrections have been mentioned.

**Table 3:** Spelling correction of lexicons.

English	Transliterated Roman Urdu	Correction
Pursuit	Hsol	Hasool
Push	Dhka	Dhakka
Receipt	Rsid	Raseed
Rectify	Islh	Islah
2. *Translation Correction:* The entire list of NRC lexicons depends on Google translator since there are certain terms for which there is no recognized or widely used Urdu equivalent. For instance, the word “acuity” is rendered as “tekshanta,” which Pakistani Urdu people are not familiar with. It could be “taiz nigah,”“taizi,” or “nokeela pan.” Another issue is that some terms were not translated, even though there are several online dictionaries that contain the Urdu translation for the same. Each translation is cross-checked with the renowned Urdu websites www.urdupoint.com, www.hamariweb.com, and www.urdu2eng.com for the purpose of translation correction, and appropriate modifications have been made to all datasets. Table 4 provides a brief list of these terms.

**Table 4:** Missing or inappropriate translation.

English	Roman Urdu translation	Corrected translation
Acrobat	Acrobat	Bazigar
Adhesion	Asanjan	Chupakna
Affiche	Affiche	Ishtehar
Alcove	Alcove	Taqqcha
Annular	Kondalkaar	Halqadaar
Ascetic	Suniyasi	Abid
Astigmatism	Astigmatism	Kajnazri
Lynch	Lunch	Maar peet
Pathetic	Dilkush	Dilsoz
3. *Cultural Emotion Correction and New Words Addition:* Due to language barriers caused by cultural variations, Pakistanis may have distinct reactions to words like “champagne,”which in Urdu means “sharab,” or “pork,” which means “soor ka gosht.” People could experience negative feelings instead of neutral ones since these are forbidden. We have included terms like “mashallah” since they are often used in Muslim culture and always convey a pleasant meaning. These adjustments aid in the accurate and effective annotation of a large volume of data. Table 5 shows a brief list of these terms.

**Table 5:** Prevailed or cultural terms for expressing emotions.

Added words	Possible English tanslation	Emotions
Alhamdulillah	Thank God	Anticipation
Mashallah	Whatever God wills	Joy
La hawl	No power	Disgust
Inshallah	If God wills	Anticipation
4. *Synonyms or Multiple Meanings Adjustment:* In the NRC emotion lexicon list, the same meanings have been provided for various English words and lexicons, which causes many other words that are used in daily life conversations to be lost. Furthermore, when people chat on social networks, it can be observed that some individuals start with some topic and other people give opinions to elaborate on the issues, but all may use different synonyms to explain the topic. For instance:
   Id1: mujhey jang o jadal pasand nahi (I don't like wars)
   Id2:Yes me too not like battles
   Id3:sahi kaha India Pakistan ki war sey kia mila kisi ko?
   Id4:han bus marka aria ka shoq hey.

In the above conversation, all four Ids are discussing warfare, so it is required to add all these words instead of a single translation, which is “jang” . A few examples have been mentioned in Table 6 to show the provided synonyms

**Table 6:** Avoiding single translation for multiple words.

English word	Google translation	Corrected synonyms
Marble	Sang e marmar	Sang e marmar
Marbles	Sang e marmar	Bachon ka khail
Meditative	Muraqba	Ghor o fikar
Meditation	Muraqba	Muraqba
Endurance	Brdasht	Brdasht
Endure	Brdasht	Zabt kerna
Awake	Baidaar	Jaagna
Awaken	baidaar	Baidaar

After correcting and validating the list of Roman Urdu emotion lexicons, text annotation has taken place. The major issue was that users of social networks do not bother about spelling while having a conversation. As a result, in order to identify emotion using the emotion lexicon, it will first be necessary to determine the degree to which the emotion lexicon and terms that denote emotion in processed text are similar.

1. *Distance estimation between words:* It has been observed that during chatting or messaging, people used to write misspelt or short-spelled wording instead of full form, for example, “phylana” or “phlana” instead of “phelana” (spread). While sometimes text is written with different start characters, like Imtehaan, “Emtehaan,” or “Amtehaan,” intentionally or unintentionally, this issue hinders the process of annotation, so we have considered similarity checking for the emotion lexicons extracted from processed sentences. For efficient labeling, a sub-list is extracted in which each word starts with the same character as the word to be evaluated as the emotion lexicon. Then a unigram is compared with each word in the sub-list, and a distance is calculated. The whole process has been described in the given algorithm, and Table 7 shows the results of character-based and term-based distance estimation or similarity.

**Table:** 

**Algorithm:**
Sort(ListofLexicons) SubList=ExtractSubList(Find all words start with same letters from sorted list) For each w_i_ in CleanText For each w_L_ in Sublist If(Distance(w_i_,w_L_) >= 0.90) return w_L_

**Table 7:** Term based and character based string similarity.

Term with English translation	Possible short spellings	Character based similarity	Term based similarity
Imtehaan (Exam)	‘emthan’	71% (Imtehaan)	90% (Imtehaan)
	‘imtahn’	71% (Imtehaan)	90% (Imtehaan)
Ghussa (Angry)	‘gssa’	80% (Ghussa)	90% (Massage)
	Ghssa	99% (Ghussa)	94% (Ghussa)
Takhfeef (Reduction)	Thkfeef	80% (Takhfeef)	71% (Takhfeef)
	Tkhfif	75% (Takhfeef)	65% (Takhfeef)

2. *Unigram features:* In order to analyze the raw data and annotate the dataset with emotion lexicons, unigram features are supposed to be more pertinent. We have constructed an emotion lexicon dictionary in both Roman Urdu and English. By identifying the obvious words and correlating them with emotion lexicons, textual data has been annotated as a baseline method. The following gives an overview of the entire process:

Corpus C can be a collection of documents, tweets, or sentences. Each sentence will then be broken down into meaningful words after the removal of useless words.

$$C=\sum_{1}^{n}D_{i}=\sum_{1}^{n}S_{i}$$

$$S_{k}=\sum_{j=1}^{m}w_{j}$$

Where *k* ϵ 1…*n* and in each sentence there are m words. In Table 8, a sample annotation for baseline purposes has been given in which sentences, useful words, emotion lexicons, and emotion tagging can be seen.

**Table 8:** Annotations of raw text.

S.No	Raw sentence	Lexicon	Emotion	Polarity	Mood
1	Correctness k se pata chaley? (how to know the correctness?)	Correctness (en)	Trust	Positive	Good
2	Wah kya baat likhi (What he wrote!)	Baat (ru)	Neutral	Neutral	Moderate
3	Tm behas mat karo na! (Do not argue!)	Behas (ru)	Anger	Negative	Bad
4	Kia husan e ittefaq hy bhai. (what a coincidence brother.)	Husn e ittefaq (ru)	Surprise	Positive	Good
5	Apne Yadgar Lamhat ko Shandar Banain (Make your memorable moments wonderful)	Shandar (ru)	Anticipation	Positive	Good

## Limitations

Not applicable.

## Ethics Statement

The data has been collected from Twitter following the polices imposed by the platform and we confirm that it is fully annonymized.

## CRediT authorship contribution statement

**Asia Samreen:** Conceptualization, Methodology, Writing – original draft. **Syed Asif Ali:** Resources, Supervision, Writing – review & editing.

## Data Availability

Emotions Lexicons in Roman Urdu, along with Emotion Polarity and Mood categorization (Original data)
Annotated data by using Roman-Urdu Emotion lexicons (Reference Data)
